# Effects of He-D Interaction on Irradiation-Induced Swelling in Fe9Cr Alloys

**DOI:** 10.3390/ma14216669

**Published:** 2021-11-05

**Authors:** Haibiao Wu, Zhen Wang, Te Zhu, Qiu Xu, Baoyi Wang, Detao Xiao, Xingzhong Cao

**Affiliations:** 1 School of Nuclear Science and Technology, University of South China, Hengyang 421001, China; wuhaibiao@163.com (H.W.); zhenwang@ihep.ac.cn (Z.W.); 2 The First Affiliated Hospital, Hengyang Medical School, University of South China, Hengyang 421001, China; 3 Multi-Discipline Research Center, Institute of High Energy Physics, CAS, Beijing 100049, China; wangboy@ihep.ac.cn; 4 Institute for Integrated Radiation and Nuclear Science, Kyoto University, Osaka 5900494, Japan; zhute@ihep.ac.cn (T.Z.); xu.qiu.8z@kyoto-u.ac.jp (Q.X.); 5 Department of Mechanical Engineering, The University of Hong Kong, Pokfulam Road, Hong Kong, China

**Keywords:** helium/deuterium, nanoclusters, positron annihilation spectroscopy, irradiation damage

## Abstract

The atomic-scale defects such as (deuterium, helium)-vacancy clusters in nuclear energy materials are one of the causes for the deterioration of the macroscopic properties of materials. Unfortunately, they cannot be observed by transmission electron microscopy (TEM) before they grow to the nanometer scale. Positron annihilation spectroscopy (PAS) has been proven to be sensitive to open-volume defects, and could characterize the evolution of the size and concentration of the vacancy-like nanoclusters. We have investigated the effects of He-D interaction on the formation of nanoscale cavities in Fe9Cr alloys by PAS and TEM. The results show that small-sized bubbles are formed in the specimen irradiated with 5 × 10^16^ He^+^/cm^2^, and the subsequent implanted D-ions contribute to the growth of these helium bubbles. The most likely reason is that helium bubbles previously formed in the sample captured deuterium injected later, causing bubbles to grow. In the lower dose He-irradiated samples, a large number of small dislocations and vacancies are generated and form helium-vacancy clusters with the helium atoms.

## 1. Introduction

FeCr alloy is the basis of reduced activation ferritic/martensitic (RAFM) steels such as F82H [1], JLF-1 [2], EUROFER97 [3], 9Cr-2WVTa [4], and CLAM [5]. Such steels are candidate structural materials for future nuclear reactors and possibly for long-term spallation neutron sources used for transmutation of spallation products. They have the advantages of low activation, excellent void-swelling resistance, and suppressed hydrogen/helium embrittlement. Unfortunately, the high neutron flux will cause the continuous production of both hydrogen and helium in the steels within the reactor via (n, p) and (n, α) transmutation reactions. As a result, swelling and embrittlement of the materials can occur by the accumulation of hydrogen and helium and the supersaturation of vacancies that build up during irradiation [6,7,8].

Reported works indicate that hydrogen isotope and helium atoms could interact with irradiation-induced vacancy defects, and they could also migrate and aggregate to form (H/He)-vacancy clusters or bubbles, which might cause undesired changes in the material properties [9,10]. The effects of helium and hydrogen on the formation of vacancy clusters have been intensively studied both experimentally and theoretically. Lee et al. suggested that the steels received more severe damage under dual ion (He and H) irradiation than under a single ion (He or H) situation [11]. The defect types in CLAM steel after co-implantation with He/H ions were summarized by Xin et al. [12]. It has been pointed out that both He- and H-vacancy clusters were formed in CLAM steel with pre-implanted He and H, but He-H-vacancy complexes were only formed in the CLAM steel that was pre-implanted with He. In order to clarify the He-D effect on hydrogen isotope retention and irradiation-induced swelling, a variety of irradiation experiments on advanced steel using plasma devices or ion beams were carried out. Recently, the research result from Zhou et al. [13] found that He injected into the bulk of RAFMs can suppress D retention whereas D behavior is not significantly affected by the damages from high-energy Fe ion, and furthermore, when the damage layers are shifted to the near surface, D retention is enhanced by both He and Fe pre-irradiation. Despite the strong research efforts aimed at the understanding of the phenomenon [14,15,16,17,18,19,20], a unified microcosmic mechanism has not emerged because of the discrepancies between experiment and theory [21]. The FeCr model alloy system is considered to be one of the most suitable systems for studying the hydrogen/helium behavior because the simplified model can reduce the influence of other elements in RAFM steel. In this work, we continue to explore the effect of He-D interaction on irradiation-induced swelling by inducing He damages in the surface of an Fe9Cr model alloy. Furthermore, we would like to generalize the characterization method of positron annihilation spectroscopy, which is not only useful in metal materials, but also useful in the study of micro-nano scale defects in 2D materials and semiconductor devices.

## 2. Experimental Procedure

**Figure 1:**
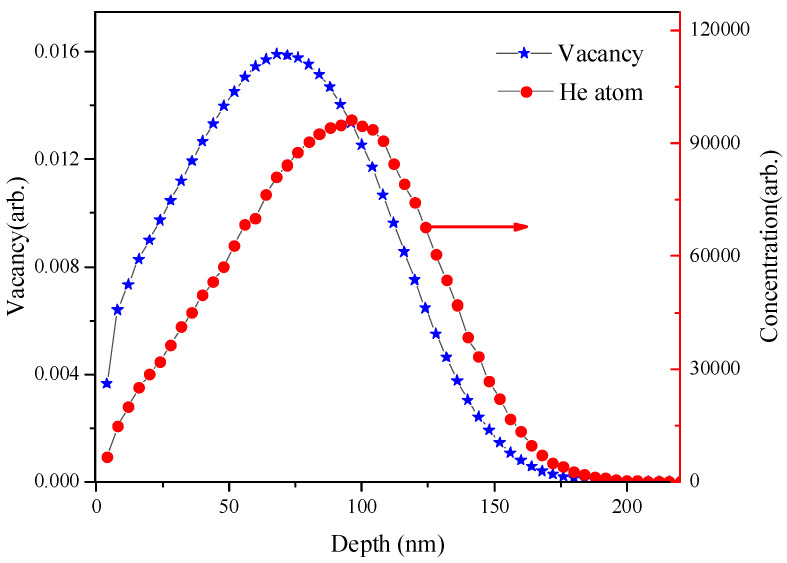
Depth profile of damage events and atoms distribution for 20 keV He-ions irradiated Fe9Cr alloy, calculated by SRIM.

The binary model alloy Fe-9wt.%Cr was melted in vacuum from high purity Fe (99.99%) and Cr (99.99%) by the high-frequency induction furnace of China Iron and Steel Research Institute Group. The bulk materials were cut into 10 mm × 10 mm square plates with a thickness of 0.3 mm and then electrolytically polished to remove possible surface contamination. We used an electrolyte with a 3:1 volume ratio of acetic acid to perchloric acid. During electropolishing, the sample surface current was 0.4 A/cm^2^ and the polishing time was 15 s. A defection-free sample was obtained to eliminate the effects of defects in the sample due to processing; the samples were annealed in a vacuum (~10^−5^ Pa) at 1100 K for 2 h and then cooled in the furnace. In the case of He ions irradiation, well-annealed specimens were irradiated with 20 keV He ions at room temperature using a 200 kV accelerator. The irradiation doses are 1 × 10^15^, 1 × 10^16^, and 5 × 10^16^ He^+^/cm^2^ respectively, and the He^+^ irradiation produced damage levels of 0.02, 0.2, and 1 dpa (displacements per atom). According to the TRIM code (Figure 1), the vacancy and He atoms are mainly distributed in the region from 0 to 190 nm, and the peaks are about 62 nm and 100 nm, respectively. The He-ions pre-irradiated specimens were irradiated with 5 keV D-ions using an Omegatron gun, in which mono-energetic D-ions were collimated and mass analyzed, at a flux of 1 × 10^13^ D·cm^−2^·s^−1^ to the nominal doses of 5 × 10^16^ D·cm^−2^ and 1 × 10^17^ D·cm^−2^ at room temperature. D-ions concentration was mainly distributed in the surface region from 0 to 65 nm and peaked at ~25 nm.

The TEM observation was conducted in a field-emission-gun analytical Tecnai G2 F30 microscope (Thermo Fisher Scientific; Waltham, MA, USA). The microstructure distribution of helium-irradiated samples was characterized by slow positron measurement. The positron experimental methods were the same with previous studies [22,23,24].

## 3. Results and Discussion

**Figure 2:**
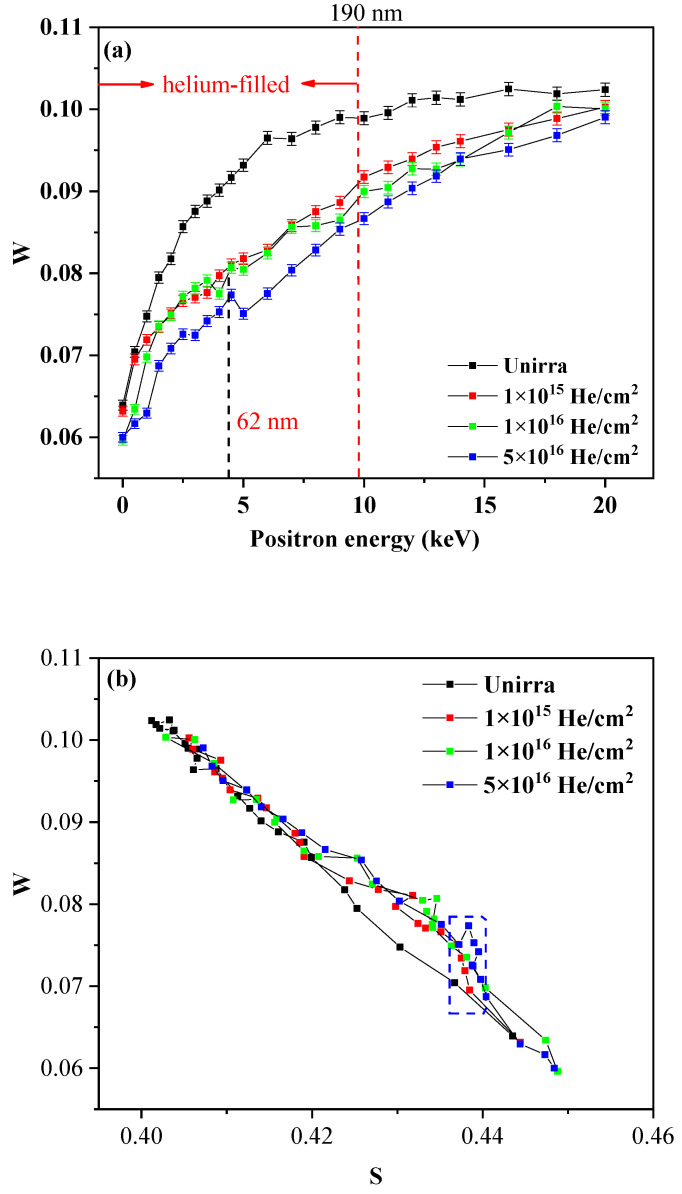
(**a**) W-E curves and (**b**) W-S curves for irradiated alloys and for unirradiated ones.

The measurement results of slow positron beam, that is, the dependence of W parameter on the implanted positron energy for the unirradiated and irradiated specimens is plotted in Figure 2a. The W parameter describing positron and orbital electron annihilation information is defined as the ratio of γ photo counts about the wing area (505.1–508.4 keV and 513.6–516.9 keV) to the total counts around 499.5–522.5 keV. When the positrons annihilate after being localized near the vacancy defects, annihilation occurs predominantly with free electrons as opposed to the defect-free or impurity atom-vacancy cluster in crystal where positrons are more likely to annihilate with the orbital electrons of lattice atoms [25,26,27,28]. Therefore, the change of W parameter (W-E and S-W plots) can reveal the structural information of the helium-defect complexes in the irradiated sample and when positrons are trapped at a large number of helium-vacancy complexes, the values of the W parameter are larger than those of empty vacancy defect. As shown in Figure 2a, the W parameters of all irradiated samples are lower than those of the unirradiated samples, because the positrons are mainly annihilated in the vacancy defects caused by helium irradiation, and the probability of annihilation with the core electrons is reduced. The W parameter of the high-dose (5 × 10^16^ He^+^/cm^2^) irradiated sample has a sudden increase at a depth of 62 nm. We speculated that this may be due to the accumulation of helium atoms in the defects caused by the irradiation and the formation of bubbles [24,29,30,31]. For the sample irradiated by helium ions, on the one hand, the concentration of helium atoms increases, and on the other hand, vacancies are also increased. If He concentration increases faster, the ratio of He/V in He-vacancy cluster will increase, resulting in the increase of W parameter. Combined with the TRIM simulation results in Figure 1, the vacancy concentration is the largest at 62 nm, and positrons are attracted by the cluster and annihilated. The proportion of helium-related information is increased in the W parameter, so a small peak is formed at 62 nm. The change in the W parameter with the S parameter can reveal the defect structure in the irradiated alloys. Figure 2b shows the effects of irradiation dose on the S-W plots for the alloys. The W and S parameters were not independent. In the irradiated alloys, the W parameter increased when the S parameter decreased, which can indicate that there is no precipitation of elements. In the previous reports, when positrons are trapped at a large number of He_m_V_n_ clusters in Fe-based alloys, the W parameter is higher than that of the empty voids because the probability of positron trapping by helium-filled clusters is low and (s, w) in S-W plots will be concentrated in a certain area. In Figure 2b, the (s, w) of the specimen irradiated with a dose of 5 × 10^16^ He^+^/cm^2^ has a significant aggregation phenomenon, which may be due to the formation of helium bubbles in the irradiated alloy.

**Figure 3:**
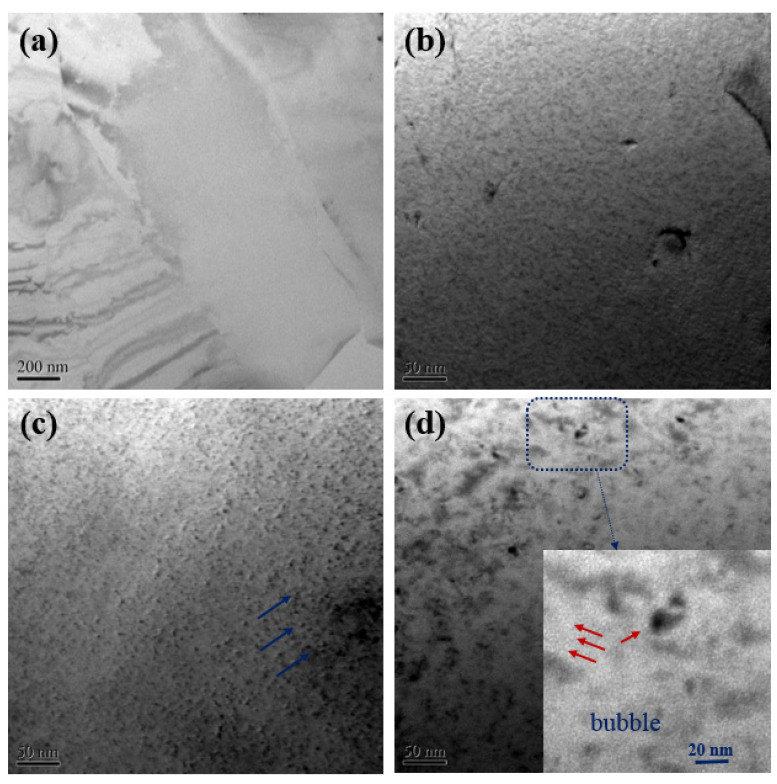
Microstructure changes of Fe9Cr alloy before and after helium irradiation at room temperature. (**a**) Unirradiated Fe9Cr, (**b**) 1 × 10^15^ He^+^/cm^2^, (**c**) 1 × 10^16^ He^+^/cm^2^ and (**d**) 5 × 10^16^ He^+^/cm^2^.

In order to confirm the formation of helium bubbles in the specimens during irradiation, the alloy samples irradiated at different doses were observed by TEM. The surface morphology of the specimens before irradiation and after irradiation is shown in Figure 3. No defects were found in the alloys before irradiation. Only a few small radiation-induced defects appeared as black spots in the alloy irradiated with a dose of 1 × 10^15^ He^+^/cm^2^, as shown in Figure 3b. As the dose increases, the black spot defects become more visible and show a three-dimensional grain shape, which were marked with blue arrows as shown in Figure 3c. These black spot defects may be small dislocation loops or defect clusters [32]. The size of these black spots in the specimen irradiated at 1 × 10^16^ He^+^/cm^2^ is significantly larger than that irradiated at low dose. The size of the black spot defect increases with the increase of the irradiation dose, which is consistent with the research results of Liu et al. They confirmed the growth of black spot defects (dislocation loops) with irradiation dose by in situ electron irradiation [33]. Small bubbles can be observed in the matrices of the Fe9Cr alloy irradiated at 5 × 10^16^ He^+^/cm^2^ (Figure 3d), while no bubbles are visible in the specimens irradiated at 1 × 10^15^ He^+^/cm^2^ and 1 × 10^16^ He^+^/cm^2^. The observed helium bubbles confirm the above-mentioned positron measurement results for the speculation that helium bubbles formed in high-dose irradiated samples.

**Figure 4:**
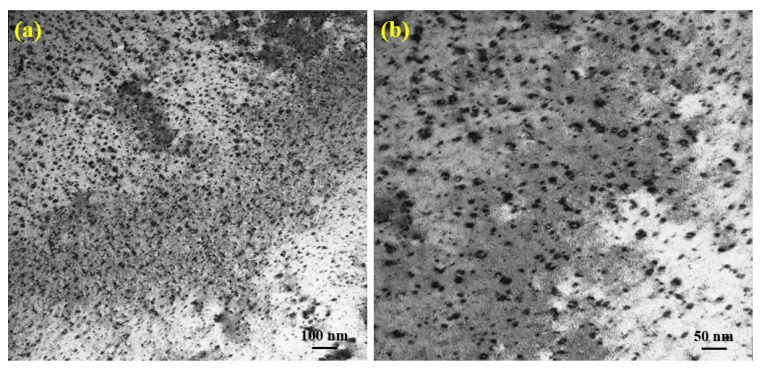
Microstructure changes of Fe9Cr alloy irradiated with deuterium at 5 × 10^16^ D/cm^2^. (**a**) Low magnification. (**b**) High magnification.

**Figure 5:**
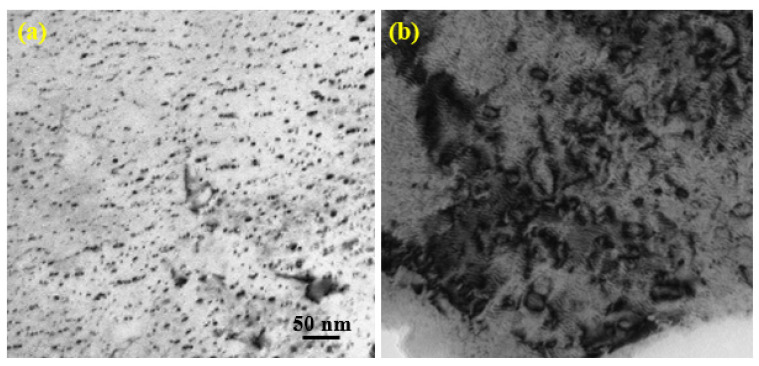
The micrographs of helium/deuterium sequential-ion irradiated Fe9Cr specimens. (**a**) 1 × 10^15^ He·cm^−2^/5 × 10^16^ D·cm^−2^ and (**b**) 1 × 10^16^ He·cm^−2^/5 × 10^16^ D·cm^−2^.

The TEM investigations of Fe9Cr specimen D-irradiated at 5 × 10^16^ D/cm^2^ are presented in Figure 4. We can see from Figure 4a,b that the microstructure in the specimen after irradiation at room temperature consisted of a high density of small dislocation loops and networks. The average size of dislocation loops induced by D-ions irradiation is 12.59 nm and the loop density is 4.2 × 10^22^ m^−3^. No bubbles were observed in the sample irradiated by D-ions. Figure 5 shows the micrographs of helium/deuterium sequential-ion irradiated specimens. The average sizes of dislocation loops induced by (1 × 10^15^ He·cm^−2^/5 × 10^16^ D·cm^−2^ and 1 × 10^16^ He·cm^−2^/5 × 10^16^ D·cm^−2^) helium/deuterium sequential-ion irradiations are 10.12 nm and 53.36 nm, respectively. The corresponding loop densities are 5.3 × 10^22^ m^−3^ and 6.7 × 10^21^ m^−3^, respectively. The average size of the loops induced by sequential-ion irradiations increased with the increase of helium irradiation dose, while the loop density decreased. The size of the dislocation loop formed in helium/deuterium sequential-ion irradiated sample was larger than that of helium single-ion irradiated specimen (see Figure 3), which indicates that the subsequent D-ions irradiation promotes the growth of the dislocation loops. No bubbles were observed in the sample irradiated by helium/deuterium sequential ion, which may be that the irradiation doses were lower and the irradiations were performed at room temperature.

**Figure 6:**
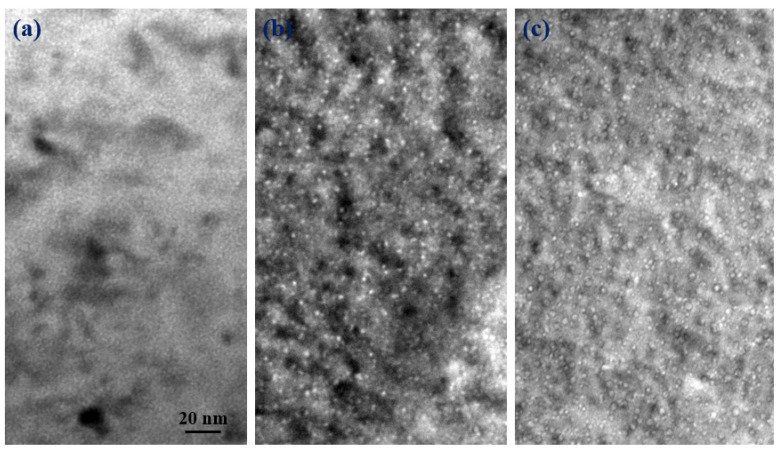
The micrographs of (5 × 10^16^ He·cm^−2^) helium single ion (**a**) and 5 × 10^16^ He·cm^−2^/5 × 10^16^ D·cm^−2^ (**b**) and 5 × 10^16^ He·cm^−2^/1 × 10^17^ D·cm^−2^ (**c**) helium/deuterium sequential-ion irradiated specimens.

**Figure 7:**
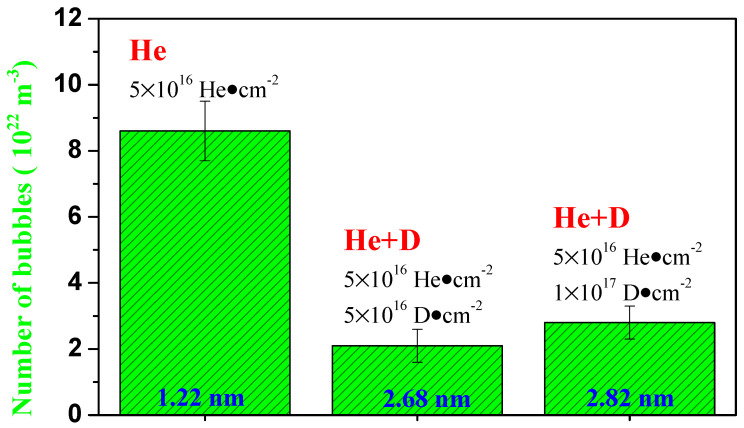
The density and average size of the bubbles in the TEM images of the irradiated samples.

Figure 6 shows the micrographs of (5 × 10^16^ He·cm^−2^) helium single-ion and (5 × 10^16^ He·cm^−2^/5 × 10^16^ D·cm^−2^ and 5 × 10^16^ He·cm^−2^/1 × 10^17^ D·cm^−2^) helium/deuterium sequential-ion irradiated specimens. For the helium single-ion irradiation, some small helium bubbles can be observed, and the density of helium bubbles was calculated to be ~8.6 × 10^22^ m^−3^, and the average size of bubbles was 1.22 nm. After the samples were irradiated by helium/deuterium sequential ion, it was found that the size of bubble became larger, indicating the growth of the bubbles was significantly promoted by the subsequent injection of D-ions. Moreover, subsequent deuterium irradiation produces a large number of vacancies, which also enhances the nucleation of the bubbles. The average sizes of the bubbles induced by (5 × 10^16^ He·cm^−2^/5 × 10^16^ D·cm^−2^ and 5 × 10^16^ He·cm^−2^/1 × 10^17^ D·cm^−2^) helium/deuterium sequential-ion irradiations are ~2.68 nm and ~2.82 nm, and the corresponding bubble densities are ~2.1 × 10^22^ m^−3^ and ~2.8 × 10^22^ m^−3^ (see Figure 7). Both the average size and density of bubbles increased with increasing deuterium irradiation dose. This is most likely because the previously formed helium bubbles in the sample captured the subsequently injected deuterium, causing the bubbles to grow. As the irradiation dose of deuterium increases, more bubbles trap deuterium and grow up, so that the average size and density of the bubbles increase at the same time.

## 4. Conclusions

The effects of He-D interaction on the formation of nanoscale cavities in Fe9Cr alloys have been studied by PAS and TEM. The main conclusions are:

1. In the lower dose He-irradiated samples, a large number of small dislocations and vacancies are generated and form helium-vacancy clusters with the helium atoms.
2. Only a few small radiation-induced defects appeared as black spots in the alloy irradiated with a dose of 1 × 10^15^ He^+^/cm^2^. As the dose increases, the black spot defects (small dislocation loops) become more visible and show a three-dimensional grain shape.
3. The microstructure in the specimen after D-ions irradiation at room temperature consisted of a high density of small dislocation loops and networks, but no bubbles were observed in the sample.
4. The size of the dislocation loop formed in helium/deuterium sequential-ion irradiated sample was larger than that of helium single-ion irradiated specimen, which indicates that the subsequent D-ions irradiation promotes the growth of the dislocation loops.
5. Nanosized bubbles are formed in the specimen irradiated with high-dose He-ions, and the subsequent implanted D-ions contribute to the growth of these helium bubbles. This is most likely because the previously formed helium bubbles in the sample captured the subsequently injected deuterium, causing the bubbles to grow.

## Data Availability

Not applicable.
